# Microanatomy of Dermal Roofing Bones in the Skull of Pipoid Frogs

**DOI:** 10.1002/jmor.70107

**Published:** 2025-12-20

**Authors:** Tomás Fornari, Johannes Müller

**Affiliations:** ^1^ Museum für Naturkunde Leibniz Institute for Evolution and Biodiversity Science Berlin Berlin Germany; ^2^ Université de Lille Villeneuve‐d'Ascq Hauts‐de‐France, Nord France

**Keywords:** frontoparietal, maxilla, microanatomy, Pipoidea

## Abstract

Previous research on bone microanatomy in amphibians suggests a correlation of microanatomical traits with both environmental and phylogenetic factors, but so far, studies had been limited to long bones. Using Pipoidea, a uniquely adapted clade of fully aquatic anurans, we investigated whether the microanatomical structures of anuran cranial bones display not only an ecological but also a clade‐specific signal. Micro‐CT scans of the skulls of five extant and three extinct pipoids species were compared with those of four phylogenetically distant, yet similarly aquatic anurans. We focused on the frontoparietal and maxillary bones, because they are among the largest bones in the anuran skull and are often preserved as fossils. From each of the bones the overall compactness, cross‐sectional area, and thickness were extracted. Statistical analysis revealed no significant difference between the three groups in thickness and cross‐sectional area, which is consistent with their shared lifestyle. Compactness, however, revealed a statistically significant difference between the Pipoidea clade and the phylogenetically distant group. Our findings suggest the presence of both a clade‐specific and an environmental signal in the bone compactness of the pipoidean skull.

## Graphical abstract

The cranial bone microanatomy in extant and extinct aquatic pipoids and phylogenetically distant aquatic anurans were analyzed. Micro‐CT data revealed no significant differences in bone thickness and cross‐sectional area among groups, that present similar aquatic lifestyles. Compactness instead showed clade‐specific values in pipoids compared to the other anurans. These findings suggest the presence of both a clade‐specific and an environmental signal in some microanatomical features in the pipoidean skull.

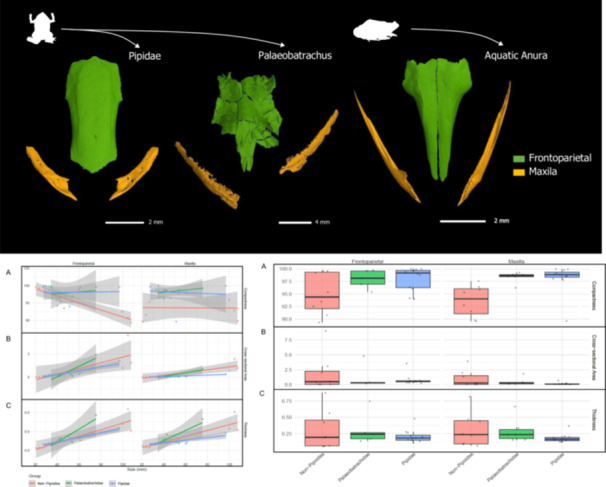

## 1 Introduction

Anurans are one of the most diverse clades of modern tetrapods, exhibiting a worldwide distribution and a variety of lifestyles. Whereas some clades display a wide range of ecological diversity, others possess a specific and unique ecology that is retained across all species, with little variation (Alexander Pyron and Wiens 2011; Wiens 2007). This is notably the case for Pipoidea, a clade whose members remain highly aquatic throughout their entire life cycle (Chipman and Tchernov 2002), which is in stark contrast to most anurans, which leave water after metamorphosis (Cannatella and Trueb 1988; Ford and Cannatella 1993; Villa et al. 2016). Members of Pipoidea possess an uncommon cranial morphology (Trueb et al. 2000), with the most distinctive characteristics being: a single, rather than paired, frontoparietal; exoccipitals that are not medially fused; absence of the premaxilla and quadratojugals, and, a reduced pars facialis and pars dentalis of the maxilla (Araújo et al. 2022; Cannatella and Trueb 1988; Roček and Veselý 1989). There is still controversy on the interpretation of these unique characters. While Maglia et al. (2001) suggest that Pipoidea retains primitive traits, other phylogenetic hypotheses (Ford and Cannatella 1993) consider them to be derived.

Previous studies indicate that bone microanatomy is largely constrained by environmental conditions, although developmental constraints may also play a role (Alfieri et al. 2021; Allemand et al. 2017; Amson et al. 2014; Houssaye et al. 2010, 2014; Klein et al. 2015; Laurin et al. 2004a). One of the major microanatomical features commonly used in these investigations is bone compactness, which usually is considered reflective of a tetrapod's lifestyle. For example, a relatively high degree of compactness is generally observed in aquatic taxa, where it contributes to buoyancy regulation, in contrast to most terrestrial species (Alfieri et al. 2021; Canoville and Laurin 2009; Etienne et al. 2025), with the exception of graviportal terrestrial species (Houssaye et al. 2016).

Thickness and cross‐sectional area are also traits which are often included in microanatomical analyses because they relate to the mechanical performance of bones under different conditions, to the overall body mass of the specimens and also, even if less directly, to the overall lifestyle (Amson et al. 2018; Dumont et al. 2013; Ebel et al. 2020; Mainland et al. 2007; Molnar 2021; Rozenblut and Ogielska 2005; Witkowska et al. 2014). Increased cortical thickness and enlarged cross‐sectional area typically provide greater resistance to compressive, tensile, bending, and torsional forces (Houssaye et al. 2016).

Furthermore, comparative studies of different clades of tetrapods such as amphibians and reptiles, suggest that bone microstructure may be associated with diagnostic phylogenetic information (Allemand et al. 2017; Legendre et al. 2014; Schoch 2014; Vidal‐García and Scott Keogh 2017; Yeh 2002). These studies are grounded in the principle that all intrinsic characters should have taxonomic value at some level in a phylogeny, including those expressed at the tissue level (De Ricqlès et al. 2004). As of today, however, research on the microanatomy of cranial bones, along with potential phylogenetic and environmental implications has predominantly focused on mammals and, occasionally, on reptiles (Mainland et al. 2007; Amson et al. 2018; Ebel et al. 2020; Amson and Bibi 2021). In contrast, the few studies of amphibians have solely investigated long bone microstructure, such as in the humerus or tibia (Canoville and Laurin 2009; Kriloff et al. 2008; Laurin et al. 2004b).

The objective of the present study is to determine whether the cranial microanatomy of Pipoidea not only reflects adaptations to an aquatic lifestyle but also exhibits a clade‐specific signal. To address this, we used X‐ray μ‐computed tomography to compare the compactness, cross‐sectional area, and thickness of the frontoparietal and maxillary bones in both extant and extinct members of Pipoidea, as well as in phylogenetically distant aquatic species. By sampling distant but ecologically similar taxa, our aim is to distinguish whether Pipoidea show distinct microanatomical patterns relative to other aquatic forms. For our case study we chose Pipoidea because their skull shows a highly specialized bone configuration, characterized by cranial traits (single frontoparietal, exoccipitals not medially fused, absence of the premaxilla, and a reduced maxilla) that are rare or absent in other anurans (Araújo et al. 2022). This distinctive morphology, combined with their predominantly aquatic ecology, lead us to examine whether the bone microanatomy of this clade also reflects its unique clade‐specific morphology.

## 2 Materials and Methods

### 2.1 Taxa

**Figure 1:**
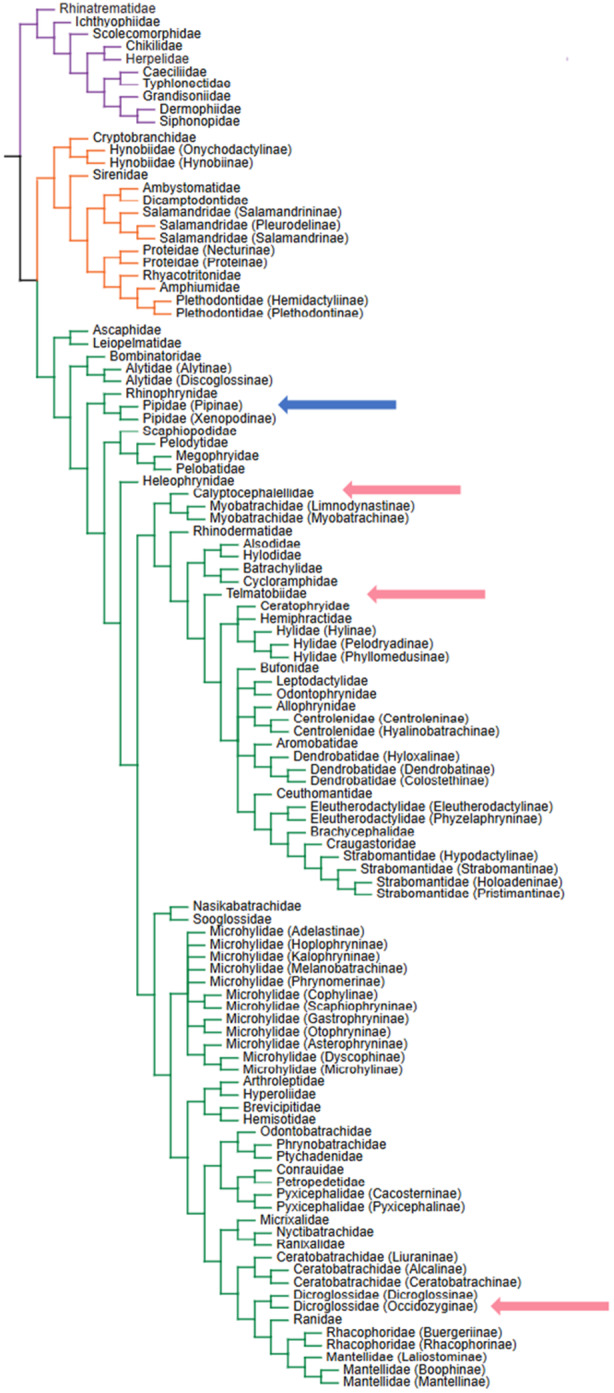
Phylogenetic tree of amphibian families. Major groups used in this study are shown in distinct colors to outline their evolutionary placement. The Pipidae group is highlighted in blue, and the phylogenetically distant comparison groups are marked in pink. Modified from Amphibia web (https://amphibiaweb.org/taxonomy/AW_FamilyPhylogeny.html).

We sampled eight fully aquatic Pipoidea species, both fossil and extant, along with four phylogenetically distant but fully aquatic anuran species, from the collections of the Museum für Naturkunde, Berlin, Germany, as well as the Martin‐Luther‐University Halle‐Wittenberg, Halle, Germany. Each species was represented by at least two specimens, except *Telmatobius macrostomus*, with an overall number of selected specimens of 26 (Table 1). With respect to extant Pipoidea, a total of five species of 41 currently recognized were selected, in order to include all five genera present in modern Pipidae (*Hymenochirus*, *Pipa*, *Pseudhymenochirus*, *Silurana*, and *Xenopus*)(Moura Gama et al. 2022). With regard to fossil Pipoidea, six specimens from the genus *Palaeobatrachus* (Palaeobatrachidae, Pipoidea), were selected for analysis (Baez et al. 2000). Proper taxonomic identification down to the species level was possible for only two (*Palaeobatrachus luedecki* and *Palaeobatrachus grandipes*), due to poor preservation. Four phylogenetically distant extant species of Pipoidea (*Telmatobius macrostomus*, *Telmatobius marmoratus*, *Occidocyga lima*, *Calyptocephalella gay*), all with a fully aquatic lifestyle, were selected for comparison purposes (Figure 1). Three groups were defined from this sampling: “Pipidae” for the extant Pipoidea species; “Palaeobatrachidae” for the extinct Pipoidea species; and “Non‐Pipoidea” for the phylogenetically distant group. Sex and age of the different anurans were not considered, as these data were missing for most specimens; however, it was at least possible to determine that all individuals examined were adults. Snout–vent length (SVL) was measured in all specimens, using a digital caliper, and was used as a proxy for overall body size (Table 1). Although some fossils were not well preserved, the snout and vent could still be reliably identified in every case with a digital caliper, allowing SVL to be recorded consistently.

**Table 1:** Studied specimens' specifications with the collection ID, Institution, Family, Genus, Species, Snout vent length (mm), Reference to the species and Morphosource ID.

ID	Institution	Family	Genus	Species	Snout vent length (mm)	Reference	Morphosource ID
ZMB_Herp_25980A	Museum für Naturkunde Berlin	Pipidae	Pipa	pipa	96,95	Linnaeus, 1758	000674725
ZMB_Herp_25980B	Museum für Naturkunde Berlin	Pipidae	Pipa	pipa	61,28	Linnaeus, 1759	000675774
ZMB_19966	Museum für Naturkunde Berlin	Pipidae	Hymenochirus	boettgeri	34,98	Tornier, 1896	000675783
ZMB_86837	Museum für Naturkunde Berlin	Pipidae	Hymenochirus	boettgeri	32,43	Tornier, 1896	000677179
ZMB_90650	Museum für Naturkunde Berlin	Pipidae	Pseudohymenochirus	merlini	40,13	Chabanaud, 1920	000677188
ZMB_90651	Museum für Naturkunde Berlin	Pipidae	Pseudohymenochirus	merlini	42,23	Chabanaud, 1920	000677204
ZMB_90652	Museum für Naturkunde Berlin	Pipidae	Pseudohymenochirus	merlini	40,11	Chabanaud, 1920	000677214
ZMB_86654	Museum für Naturkunde Berlin	Pipidae	Silurana	tropicalis	51,51	Gray, 1864	000677268
ZMB_86672	Museum für Naturkunde Berlin	Pipidae	Silurana	tropicalis	60,01	Gray, 1864	000678140
ZMB_29204	Museum für Naturkunde Berlin	Pipidae	Xenopus	laevis	27,57	Daudin, 1802	000678160
ZMB_29204A	Museum für Naturkunde Berlin	Pipidae	Xenopus	laevis	32,52	Daudin, 1802	000677295
ZMB_29204B	Museum für Naturkunde Berlin	Pipidae	Xenopus	laevis	29,75	Daudin, 1802	000677304
ZMB_18896	Museum für Naturkunde Berlin	Ceratophryidae	Telmatobius	marmoratus	53,21	Duméril and Bibron, 1841	000677277
ZMB_26211	Museum für Naturkunde Berlin	Ceratophryidae	Telmatobius	marmoratus	46,23	Duméril and Bibron, 1841	000677286
ZMB_7700	Museum für Naturkunde Berlin	Ceratophryidae	Telmatobius	macrostomus	99,88	Peters, 1873	000678149
ZMB_26117	Museum für Naturkunde Berlin	Calyptocephalellidae	Calyptocephalella	gayi	104,76	Duméril & Bibron, 1841	000678091
ZMB_4324	Museum für Naturkunde Berlin	Calyptocephalellidae	Calyptocephalella	gayi	107,61	Duméril & Bibron, 1841	000678103
ZMB_91009	Museum für Naturkunde Berlin	Ranidae	Occidocyga	lima	27,03	Gravenhorst, 1829	000678113
ZMB_91010	Museum für Naturkunde Berlin	Ranidae	Occidocyga	lima	23,78	Gravenhorst, 1829	000678122
ZMB_91011	Museum für Naturkunde Berlin	Ranidae	Occidocyga	lima	20,74	Gravenhorst, 1829	000678131
GMH_Ce_III_1312_1932	Martin‐Luther‐University Halle‐Wittenberg	Palaeobatrachidae	Palaeobatrachus	grandipes	33	Tschudi, 1839	000682955
ZMB_MBAM_878	Museum für Naturkunde Berlin	Palaeobatrachidae	Palaeobatrachus	luedecki	40	Tschudi, 1840	000678322
ZMB_AM_606	Museum für Naturkunde Berlin	Palaeobatrachidae	Palaeobatrachus	sp	34,5	Tschudi, 1841	000678363
GMH Ce IV‐6699‐1933	Martin‐Luther‐University Halle‐Wittenberg	Palaeobatrachidae	Palaeobatrachus	sp	54	Tschudi, 1841	000678347
GMH Ce IV‐6691‐1933	Martin‐Luther‐University Halle‐Wittenberg	Palaeobatrachidae	Palaeobatrachus	sp	43	Tschudi, 1841	000678337
GMH Ce III‐4962‐1932	Martin‐Luther‐University Halle‐Wittenberg	Palaeobatrachidae	Palaeobatrachus	sp	41,7	Tschudi, 1841	000678328

### 2.2 Bone Samples

We selected the frontoparietal and the maxilla for our bone microanatomy analysis because they are among the major bony elements that make up large parts of the anuran skull, and are often well‐preserved in the fossil record. These bones also are characterized by a specific morphology found in Pipidae, differing significantly from other anurans, which makes them suitable for detecting phylogenetic signals (Araújo et al. 2022; Cannatella and Trueb 1988; Yeh 2002). Moreover, frog skull anatomy has been linked to body size, feeding biology, and lifestyle (e.g., terrestrial, aquatic, fossorial), suggesting that cranial elements can reflect ecological and functional patterns (Paluh et al. 2020; Trueb et al. 1993). Focusing on the frontoparietal, which forms the roof of the braincase, and the maxilla, which composes much of the upper jaw, therefore allows us to evaluate whether cranial microanatomy captures both ecological adaptations and clade‐specific characteristics.

### 2.3 3D Reconstructions

Micro X‐ray computed tomography (μ‐CT) of the taxa was taken at the Fraunhofer Development Center X‐ray Technology EZRT (Fraunhofer IIS), using the Fraunhofer EZRT High Energy CT XXL‐CT device, and at the Museum für Naturkunde (Laboratory identification ID RRID:SCR_022585), using a Phoenix nanotom X‐ray|s tube (Waygate Technologies, Baker Hughes, Wunstorf, Germany; SCR_022582) as well as a Comet YXLON FF85 (Comet YXLON GmbH, Hamburg, Germany; Equipment identification ID RRID:SCR_020917). Scan settings depended on sample size and preservation, but typically ranged from 90 to 100 kV, 4.2–10 μm voxel size, and 50–350 μA, yielding about 2500 projections at 750 ms. The cone beam reconstruction was performed using the Nexus reconstruction software (Comet YXLON GmbH, Hamburg, Germany) and the datos|x 2 reconstruction software (Waygate Technologies, Baker Hughes, Wunstorf, Germany; datos|x 2.2), and the data were visualized in VGStudio Max 3.5 (Volume Graphics GmbH, Heidelberg Germany). The maxilla and frontoparietal were segmented manually to define the outline of the bones in each slice. Then each bone was subsequently extracted as separate objects from the rest of the skull. All the bones extracted where orientated in a standardized manner, with the x‐axis aligned with the lateral axis, the y‐axis aligned with the ventral/dorsal axis, and the z‐axis aligned with the anterior/posterior axis. The pitch (antero‐posterior tilt of the head) was set using the horizontal semicircular canal when it could be reliably identified; in specimens where this was not possible, we used the closest comparable orientation based on the best‐preserved anatomical landmarks to maintain consistency across taxa. In the case of *Palaeobatrachus luedecki*, the frontoparietal element was not preserved, and thus only the maxilla was selected.

### 2.4 Microanatomical Quantifications

Each segmented bone was exported as a stack of TIFF images representing cross‐sections perpendicular to the anteroposterior axis. The different TIFF images were imported into the software ImageJ 1.52i (https://imagej.net/ij/), and the following macro script was used: for each cross‐section, the script first converted the image into a binarized greyscale image (bone vs. background), using the threshold function to ensure comparability across specimens. However, in some fossils, the preservation prevented a reliable estimation of microanatomical parameters. In particular, diagenetic alterations and localized degradation caused the algorithm to misinterpret the bone matrix, of the binarized images of the cross‐sections, as being less compact than it actually was, occasionally producing anomalously low compactness values. To minimize bias, cross‐sections, from the segmented bones, affected by such preservation issues were excluded from the analysis.

**Figure 2:**
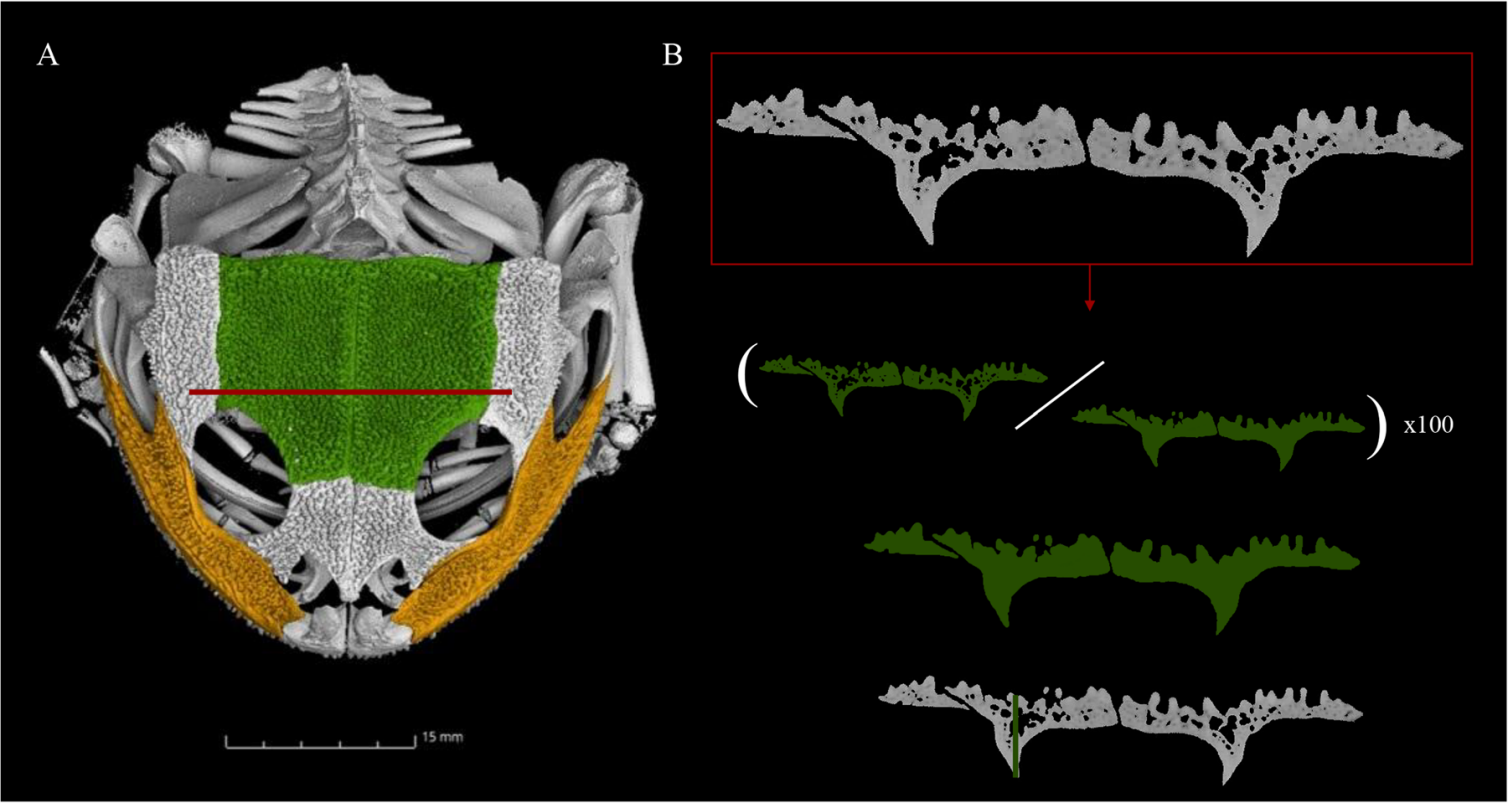
A. Micro‐CT scan of the specimen *Calyptocalicephalella* gayi ZMB 4324 with the frontoparietal and maxilla highlighted in green and yellow respectively, red line indicates middle of the frontoparietal. B. Visualization of the cross‐section of the middle frontoparietal with the visualized microanatomical parameters Compactness, Cross‐sectional Area, and Thickness.

The different microanatomical traits were estimated in each of the bones for each taxon (Figure 2). Compactness (C) was measured as the ratio between the number of bone pixels and the total number of pixels in the bone cross‐section, expressed as a percentage, and considering all cross‐sections of the same bone. The cross‐sectional area (CSA) was measured as the total surface, based on the external contours of the bone, for each slide. Lastly, thickness (Th) was measured as the longest distance between the top of the dorsal side and the bottom of the ventral side. This was processed for the entire image stack of each of the bones in every specimen.

In order to analyze the data, the R statistical software environment was used (https://www.r‐project.org/), specifically the EDI RStudio version 4.2.1 (2022‐06‐23 ucrt). To determine whether differences in microanatomical traits (compactness, cross‐sectional area, and thickness), measured in the frontoparietal and maxilla, present a correlation with the three anuran groups in this study, we first tested for normality of the data using the Shapiro–Wilk test (González‐Estrada and Cosmes 2019). As traits deviated from a normal distribution, non‐parametric methods were chosen.

Afterwards, we compared the differences in body size among the three groups using a Kruskal–Wallis test (Ostertagová et al. 2014), followed by an evaluation of the effect of body size on each trait with a linear regression for each trait–bone combination. Where size significantly influenced a trait, residuals from the regression (size‐corrected values) were used to control for allometry. Lastly, group differences in the mean trait residuals were tested using Kruskal–Wallis tests, followed by Dunn's post hoc pairwise comparisons (Dinno 2015) to identify which groups differed significantly from each other.

## 3 Results

### 3.1 Transverse Sections

**Figure 3:**
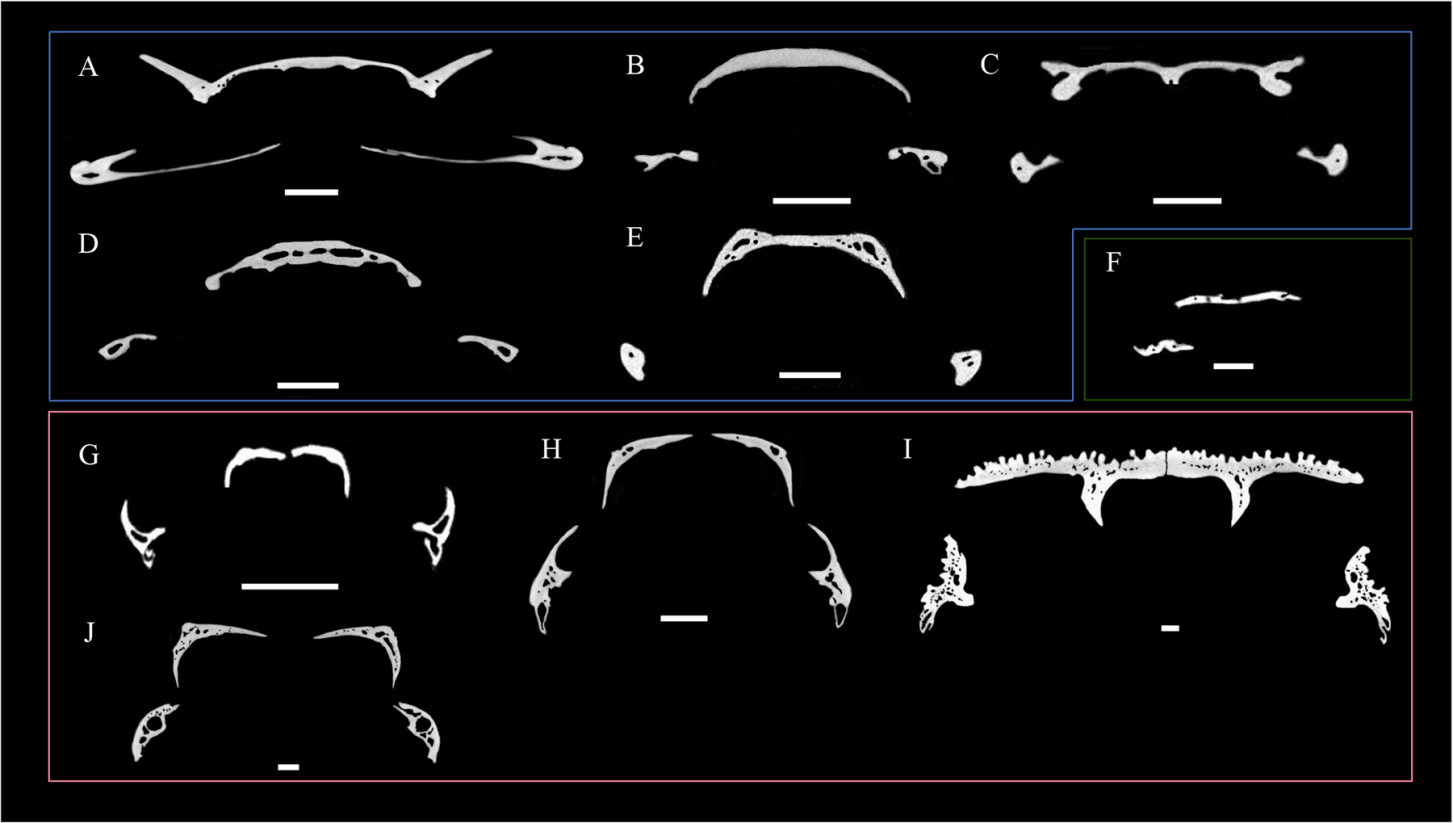
Schematic drawing illustrating the various patterns observed in the transverse sections for the frontoparietal (top) and maxilla (bottom) of each taxa. White, bone; black, cavities. A. *Pipa pipa* ZMB Herp 25980b. B. *Xenopus laevis* ZMB29204a. C. *Hymenochirus boettgeri* ZMB 19966. D. *Silurana tropicalis* ZMB86654. E. *Pseudohymenochirus merlini* ZMB 90650. F. *Palaeobatrachus* sp MB AM606 G. *Occidocyga lima* ZMB 91009. H. *Telmatobius marmoratus* ZMB26211. I. *Calyptocephalella gayi* ZMB 26117. J. *Telmatobius macrostomus* ZMB7700. Scale bar 1 mm.

In the transverse sections of Pipidae (Figure 3A–E), both the frontoparietal and maxilla exhibit an overall thin bone layer with only few vascular canals and limited trabecular development, resulting in a uniformly dense and compact osseous structure. Most taxa, such as *Pipa pipa*, *Xenopus laevis*, and *Hymenochirus boettgeri*, show only a few small vascular openings, typically restricted to the lateral margins of the bones (both in the frontoparietal and maxilla). *Silurana tropicalis* displays an exception in its microstructure within the group, displaying slightly larger internal cavities and a more developed trabecular having a more vascularized bone.

The Non‐Pipoidea group presents a microstructure different from Pipidae. Transverse sections of the frontoparietal and maxilla (Figure 3G–J) reveal more vascular canals and hollow spaces, generating a more porous bone. This pattern is consistent across all taxa in the group, particularly in the frontoparietal, though the maxilla generally shows slightly less internal porosity. The only exception is *O. lima*, whose frontoparietal is characterized by almost no vascular canals or trabeculae, resulting in a compact bone, similar to the ones observed in the Pipidae group.

For the Palaeobatrachidae (Figure 3F), the state of preservation generally limited the ability to describe their internal microstructure in detail. When transverse sections could be examined, however, they revealed tightly compacted cortical bone with almost no vascular canals, a configuration closely resembling that observed in most Pipidae.

### 3.2 Compactness Analysis

**Figure 4:**
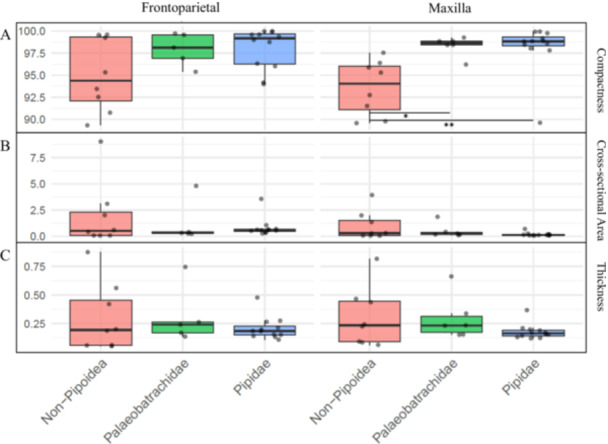
Boxplots of residuals (size‐corrected) microanatomical traits in frontoparietal (left) and maxilla (right). Traits shown are compactness (A), cross‐sectional area (B), and thickness (C). The thick black line inside each box represents the median, while the box itself spans from the first quartile to the third quartile. The whiskers line represents the minimum and maximum. Groups: Non‐Pipoidea (red), Palaeobatrachidae (green), and Pipidae (blue). Significant differences between groups marked with * for a *p* value <= 0.05 and ** for a *p* value <= 0.01.

Species of Pipidae exhibited consistently high compactness in both, the frontoparietal and the maxilla, with minimal variation along the bone lengths (Figure 4A, blue area). Mean values (Table 2) ranged from 97.8% to 99.9%, particularly in *P. pipa*, *X. laevis*, *H. boettgeri*, and the specimen 86672 of *S. tropicalis*. In contrast, Non‐Pipoidea species (Figure 4A, red area) generally displayed lower compactness, especially *C. gayi*, whose frontoparietal reached only 89%. However, *O. lima* represents an exception within this group, with compactness values (up to 99.5%) comparable to Pipidae. Palaeobatrachidae (Figure 4A, green area) showed a pattern closely resembling that of extant Pipidae, with high compactness and little variation in both bones.

**Table 2:** Summary of measurements from the macro script run in ImageJ, with mean Compactness (C%), mean Cross‐sectional Area (CSA), and mean Thickness (mm), in each bone of each specimen.

			Frontoparietal			Maxilla		
ID	Genus	Species	C (%)	Th (mm)	CSA (mm2)	C (%)	Th (mm)	CSA (mm2)
ZMB_Herp_25980A	Pipa	pipa	99.6	0.4786	3.5615	99.93	0.3697	0.6907
ZMB_Herp_25980B	Pipa	pipa	99.58	0.2129	1.0443	97.8	0.1704	0.242
ZMB_19966	Hymenochirus	boettgeri	99.35	0.1511	0.512	99.93	0.1542	0.07595
ZMB_86837	Hymenochirus	boettgeri	98.74	0.1539	0.5409	98.59	0.1586	0.07211
ZMB_90650	Pseudohymenochirus	merlini	96.01	0.1975	0.5627	98.44	0.2093	0.1306
ZMB_90651	Pseudohymenochirus	merlini	96.37	0.1842	0.5935	98.85	0.1891	0.11
ZMB_90652	Pseudohymenochirus	merlini	94.17	0.1844	0.6184	98.81	0.1981	0.1173
ZMB_86654	Silurana	tropicalis	93.98	0.276	0.6915	89.62	0.1686	0.1872
ZMB_86672	Silurana	tropicalis	99.68	0.2603	0.6271	98.03	0.1429	0.1486
ZMB_29204	Xenopus	laevis	99.98	0.1316	0.3268	99.76	0.1313	0.07446
ZMB_29204A	Xenopus	laevis	99.88	0.1408	0.3523	99.16	0.1235	0.08625
ZMB_29204B	Xenopus	laevis	99.96	0.1087	0.2611	98.87	0.1203	0.07323
ZMB_18896	Telmatobius	marmoratus	95.33	0.2002	0.5813	97.54	0.2436	0.2882
ZMB_26211	Telmatobius	marmoratus	93.44	0.1875	0.4121	96.39	0.2244	0.2641
ZMB_7700	Telmatobius	macrostomus	92.52	0.562	2.0014	95.88	0.4667	1.3487
ZMB_26117	Calyptocephalella	gayi	90.76	0.8761	9.0181	92.74	0.8176	3.9276
ZMB_4324	Calyptocephalella	gayi	89.33	0.4215	3.0867	89.78	0.4377	1.976
ZMB_91009	Occidocyga	lima	99.55	0.0609	0.05743	95.7	0.06215	0.03468
ZMB_91010	Occidocyga	lima	99.27	0.05829	0.05518	91.51	0.08481	0.05612
ZMB_91011	Occidocyga	lima	99.58	0.0512	0.05926	89.58	0.09274	0.05771
GMH_Ce_III_1312_1932	Palaeobatrachus	grandipes	99.55	0.7495	4.8245	99.25	0.6634	1.8454
ZMB_MBAM_878	Palaeobatrachus	luedecki	—	—	—	95.42	0.2722	0.24
ZMB_AM_606	Palaeobatrachus	sp	99.71	0.1361	0.3108	98.83	0.152	0.1573
GMH Ce IV‐6699‐1933	Palaeobatrachus	sp	96.09	0.2598	0.3921	98.53	0.3369	0.406
GMH Ce IV‐6691‐1933	Palaeobatrachus	sp	97.03	0.1663	0.2163	98.89	0.1534	0.1027
GMH Ce III‐4962‐1932	Palaeobatrachus	sp	98.12	0.2403	0.2561	98.68	0.233	0.1662

**Figure 5:**
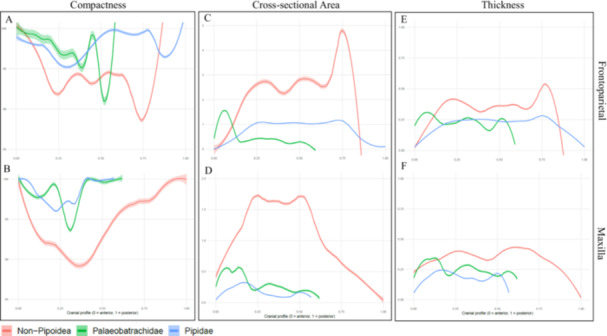
Mean traits values of compactness (A,B), cross sectional area (C,D) and thickness (E,F), across the cranial profile of each group. Regressions lines with a 95% confidence interval (shaded area) represent each of the group means, Non‐Pipoidea (red), Palaeobatrachidae (green), and Pipidae (blue).

When compactness was examined along the length of the bones using the mean values (Figure 5A,B), Pipidae consistently exceeded Non‐Pipoidea and Palaeobatrachidae across most positions along the profile. Both Pipidae and Non‐Pipoidea displayed a decline in compactness in the anterior half and posterior end of the frontoparietal, while in the maxilla this decrease occurred mainly anteriorly. Palaeobatrachidae followed a similar trend but exhibited a pronounced mid‐shaft decrease not seen in the extant groups.

However, when these trends are examined statistically, the separation between groups becomes less straightforward. The Kruskal–Wallis test (Table 3) revealed no significant differences in compactness among groups for the frontoparietal (*p* = 0.105), whereas the maxilla showed a significant difference (*p* = 0.00197). Given the results, the Dunn's post hoc test (Table 4) was performed only for the compactness in the maxilla between different groups, which revealed that Non–Pipoidea were different from both Pipidae (*p* = 0.040) and Palaeobatrachidae (*p* = 0.0018), while the latter two groups do not differ statistically from each other (*p* = 0.647).

**Table 3:** Results of Kruskal–Wallis tests for each of the microanatomical characteristics (compactness, cross‐sectional area, and thickness) across different bones (frontoparietal and maxilla).

Kruskal‐Wallis test					
Trait	Bone	“n”	“statistic”	“df”	“p”
Compactness	Frontoparietal	26	4.5083077	2	0.105
Compactness	Maxilla	26	12.459402	2	0.00197
Cross‐sectional Area	Frontoparietal	26	1.4230769	2	0.491
Cross‐sectional Area	Maxilla	26	2.767094	2	0.251
Thickness	Frontoparietal	26	0.4961539	2	0.78
Thickness	Maxilla	26	2.3931624	2	0.302

*Note:* Values are sample size (*n*), test statistic, degrees of freedom (*df*), and p‐value. Significant results (*p* < 0.05) are shown in bold.

**Table 4:** Results of Dunn's post hoc tests for differences among groups (NP = Non‐Pipoidea, Pal = Palaeobatrachidae, Pip = Pipidae) for the compactness in the maxilla.

Dunn test				
Trait	Bone	groups	p. adj	signif
Compactness	Maxilla	NP ‐ Pal	0.0404	*
Compactness	Maxilla	NP ‐ Pip	0.00184	**
Compactness	Maxilla	Pal ‐ Pip	0.647	ns

*Note:* Significance levels: *p* < 0.05 (*), *p* < 0.01 (**), ns = not significant.

### 3.3 Cross‐Sectional Area Analysis

In Pipidae the values of both bones, expressed in mm^2^, exhibited a minimal variation of the overall cross‐sectional area (Figure 4B, blue area). Looking at the mean values (Table 2), *P*. *pipa* presented by far the highest cross‐sectional area, with values around 3.56 mm^2^ for the frontoparietal and 0.69 mm^2^ for the maxilla. In contrast, Non‐Pipoidea exhibited a far more heterogeneous pattern (Figure 4B, red area), apparently displaying a higher cross‐sectional area and a larger variation in both the frontoparietal and the maxilla. Comparing the mean values for either bone (Table 2), *T. marmoratus*, and *O. lima* presented relatively low values of 0.288 mm^2^ and 0.034 mm^2^, while *T. macrostomus* and *C. gayi* showed the opposite, with values reaching up to 9.01 mm^2^ and 3.92 mm^2^. Palaeobatrachidae (Figure 4B, green area) largely resembled Pipidae, maintaining consistently low values, with the exception of *P. grandipes*, which exhibited higher overall cross‐sectional areas in both bones, measuring 4.824 mm² and 1.845 mm².

When these values were examined along the length of each bone (Figure 5C,D), Non–Pipoidea presented mean values that appear higher than the two other groups, with a characteristic plateau in the mid‐region of the bones. In contrast, Pipidae and Palaeobatrachidae showed similar profiles and ranges of mean values. However, Palaeobatrachidae was marked by a pronounced increase in the anterior region of both bones, whereas Pipidae maintained an overall stable range of values.

As with compactness, these qualitative trends are not fully reflected in the statistical analyses. The Kruskal–Wallis test (Table 3) revealed no significant differences among groups for either bone (frontoparietal: *p* = 0.491; maxilla: *p* = 0.251). Given these outcomes, no Dunn's post hoc test was performed.

### 3.4 Thickness Analysis

The thickness, expressed in mm, observed in Pipidae presented minimal variation in both frontoparietal and maxilla (Figure 4C, blue area). Based on the mean values (Table 2), *P. pipa* presented by far the highest thickness (around 0.478 mm for the frontoparietal and 0.369 mm for the maxilla). In contrast, Non–Pipoidea exhibited both higher overall thickness and markedly greater variability (Figure 4C, red area). With in this group, *T. macrostomus* and *C. gayi* showed the highest mean values for either bone (0.876 mm and 0.562 mm in the frontoparietal, and 0.817 mm and 0.4667 mm in the maxilla, respectively), whereas *O. lima* presented the smallest thickness values (0.0512 mm in the frontoparietal and 0.0621 mm in the maxilla). Palaeobatrachidae, displayed thickness values comparable to those of Pipidae (Figure 4C, green area).

When looking at the mean thickness across the length of the two bones, Non–Pipoidea presented slightly higher values along the profile (Figure 5E,F), with a plateau in the central region of the frontoparietal. Similar to the cross‐sectional area, both Pipidae and Palaeobatrachidae showed analogous profiles and ranges of values. However, Palaeobatrachidae was marked by a pronounced increase in the anterior region of both bones, whereas Pipidae maintained an overall stable range of values.

As seen in the previous microanatomical elements, when the qualitative trend is examined statistically the results are not the same. As a matter of fact, the Kruskal–Wallis test (Table 3) revealed no significant differences among groups in both bones (frontoparietal: *p* = 0.78; maxilla: *p* = 0.302). Given the results, the Dunn's post hoc test was not performed.

## 4 Discussion

### 4.1 Environmental Implications of Bone Microanatomy

Our findings provide the first analysis of cranial bone microanatomy of Pipoidea, which can be characterized by highly compact skull bones combined with a low cross‐sectional area and reduced thickness (Figure 4).

When examining compactness specifically, all taxa analyzed in this study showed relatively high compactness values compared to those reported for animals with different lifestyles (Alfieri et al. 2021; Amson and Bibi 2021; Etienne et al. 2025; Kalita et al. 2022; Konietzko‐Meier et al. 2018; Laurin et al. 2004a). High compactness has been consistently associated with buoyancy control in aquatic vertebrates (Canoville and Laurin 2009; Kondo et al. 2023; Konietzko‐Meier et al. 2018; Kriloff et al. 2008). The compactness results of our analysis (Figure 4A) are consistent with patterns commonly reported for aquatic taxa, which frequently exhibit increased bone density across different skeletal elements. Although most studies focus on long bones and only a limited number address cranial dermal bones, the relatively high compactness observed here is in line with the functional expectations for species with an aquatic lifestyle. Furthermore, the higher compactness observed in Pipoidea may not be driven only by ecology; developmental factors may also play an important role in their ossification (Yeh 2002). Their flat, hyperossified frontoparietals and reduced maxillae (supplementary online material Figure 1) create a more uniform and dense bone structure (Cannatella and Trueb 1988; Yeh 2002), which could limit the development of spongy bone, promoting a more uniform ossification.

In tetrapods, high cross‐sectional area values are usually associated with robust bones and increased body mass (Ebel et al. 2020; Mielke et al. 2018; Ryan and Shaw 2013); some studies have interpreted this trait, in long bones, as another aquatic adaptation due to its contribution to bone density (Kriloff et al. 2008; Laurin et al. 2004a; Mazin 2001). Comparably, in our dataset, aquatic taxa consistently exhibited relatively smaller cross‐sectional area values in the cranial bones examined (Figure 4B), a pattern we interpret to be consistent with the generally small skulls and slender cranial architecture characteristic of many pipids (supplementary online material Figure 1). However, *P. pipa*, *P. grandipes*, *T. macrostomus* and *C. gayi* stood out for their particularly high cross‐sectional area values (Table 2), which we attribute to their comparatively large size (Watson et al. 2017). In *P. pipa* and *P. grandipes*, elevated values can likely be explained by their much larger body size relative to other members of Pipidae: even though their skull bones remain flat, the frontoparietal and maxilla contribute to a larger cross‐sectional area. Consequently, in these cranial bones, unlike in long bones, cross‐sectional area may primarily reflect overall body size rather than more ecological factors.

Bone thickness has been shown to provide valuable insights into both tetrapod lifestyle and bone functional properties (Biknevicius et al. 1993; Ebel et al. 2020; Laurin et al. 2004b). Our results showed overall low thickness in Pipoidea (Figure 4C), a pattern that could potentially relate with the overall flat bones present in pipids that develop during the early stages of metamorphosis, limiting subsequent bone growth (Yeh 2002).

When observing the function of the maxilla in Pipoidea it is possible to see that it contributes little to their feeding mechanics. Because pipids lack both tongue and teeth, they rely less on the jaws and more on their forelimbs and a hyobranchial pump to capture prey (Vogt et al. 2017). This reduced functional demand, together with the reduced development of the maxilla, may be reflected in the bone's microanatomy, with consistently low thickness in both extant Pipidae and the fossil Palaeobatrachidae. A comparable pattern is also present in *T. marmoratus* and *T. macrostomus*, which show low average maxillary thickness (Table 2) and share similar developmental trajectories and feeding strategies (Barrionuevo 2016). By contrast, these patterns differ from those in long bones, where high cortical thickness is commonly associated with diving and underwater locomotion (Laurin et al. 2004b). Further research will be necessary to determine whether dermal bone thickness reflects ecological adaptations in a way similar to long bones.

### 4.2 Clade‐Specific Values

Previous studies suggested that at least some characters from bone microanatomy can provide a strong phylogenetic signal (Cubo et al. 2005; Laurin et al. 2004b). Our findings support such a relationship, especially for the maxilla bone compactness. When comparing Pipoidea with other aquatic anurans, compactness values in the maxilla remained significantly different, despite all taxa living in similar aquatic environments. Furthermore, there was no statistical difference in the maxilla compactness between extant pipids and fossil Palaeobatrachidae (Table 4), suggesting that Pipoidea exhibit clade‐specific compactness values of the maxilla throughout their evolutionary history.

The clade‐specific patterns observed in the maxilla among Pipidae may reflect underlying developmental constraints. The ancestral condition in vertebrates, including salamanders and caecilians, is for jaw ossification to occur early; however, delayed jaw ossification is a synapomorphy of Anura (Yeh 2002). Interestingly, Pipoidea show an early ossification of the jaw, including the maxilla, which reverts to the ancestral vertebrate condition (Ford and Cannatella 1993; Maglia et al.^2001^; Yeh 2002). This shift may underlie the compact morphology of the maxilla in both fossil and extant members of the clade, reinforcing its value as a phylogenetically informative trait.

In contrast, cross‐sectional area and thickness showed a different pattern. All investigated groups displayed no significant results between the different bones and microanatomical characters. This indicates that cross‐sectional area and thickness may be more strongly shaped by species‐specific developmental or ecological factors and less constrained by a clade‐specific range of values.

## 5 Conclusions

The relationship between microanatomical traits and phylogeny and environment has been almost exclusively studied in amphibian long bones. Our study provides the first comprehensive assessment of cranial bone microanatomy in Pipoidea, demonstrating that also dermal bones may be useful for analyzing lifestyle and environmental adaptations. Our findings show that microanatomical characteristics in Pipoidea vary across the group in ways that reflect both ecological adaptations and clade‐specific patterns. Taken together, our results indicate that while cranial bone compactness may be a combination of both functional demands and phylogenetic history, variation in thickness and cross‐sectional area likely reflects a complex interplay of ecological and developmental factors.

## Author Contributions

**Tomás Fornari:** methodology, software, data curation, formal analysis, writing – original draft. investigation, conceptualization. **Johannes Müller:** funding acquisition, investigation, project administration, resources, supervision, validation, writing – review and editing.

## Conflicts of Interest

The authors declare no conflicts of interest.

## Supporting information

Supplementary_text.

Table.

Figures.

Figures_legends.

## Data Availability

The data set of all micro‐CT stack images is available from MorphoSource (MorphoSource IDs in Table 1).
