# Supplementary material for: Microanatomy of Dermal Roofing Bones in the Skull of Pipoid Frogs
Source: J Morphol. 2025 Dec 20;286(12):e70107. doi: 10.1002/jmor.70107 (PMC12717838; doi:10.1002/jmor.70107)
Supplement: Supplementary file 1 — Supplementary_text. [file JMOR-286-e70107-s004.pdf]

Supplementary text

External bone morphology

Pipidae

The frontoparietal within Pipidae reveals an overall low level of interspecific variation (Supplementary fig. 1), consisting of a single, straight, and dorsally concave bone that bears an extended supraorbital crest and a poorly developed posterior median process. In *Pipa pipa* (Supplementary fig. 1C, D), the bone is characterized by a well-developed three-point incision into the nasal, while *Xenopus laevis* and *Silurana tropicalis* (Supplementary fig. 1H-L) show a V-shaped depression. *Pseudhymenochirus merlini* (Supplementary fig. 1E-G) exhibits a frontoparietal that extends laterally into the anterior region of the skull and is fused with the nasal. The maxilla also exhibits minimal interspecific variation, present on either side of the skull with an arc-shaped curve with a pronounced pars palatina (Supplementary fig. 1). In the maxilla of *Hymenochirus boettgeri* (Supplementary fig. 1A, B), the pars palatina expands laterally into a small area around the orbital, while the pars facialis marks the connection with the nasal and the squamosal.

Non-Pipoidea

In all taxa of Non-Pipoidea (Supplementary fig. 2), the frontoparietal is made up of two paired structures that are separated by a median suture. These structures are expanded posteriorly, possess a rounded paraoccipital process and a marked posterior median process, having a straight and elongated shape. *T. marmoratus* (Supplementary fig. 2A, B) exhibits a median suture that does not run across the entire bone, thus creating a forked structure that extends anteriorly. In the frontoparietal of *C. gayi* (Supplementary fig. 2F, G), the dorsal surface of the bone is strongly ornamented. As for the maxilla, every pars is present and easily recognizable (Supplementary fig. 2), unlike in Pipoidea. The maxilla of *O. lima* (Supplementary fig. 2D, E, H) does not present the typically observed arc-shaped morphology, but rather a more linear structure, with a dominant presence of the pars palatina. The maxilla of *C. gayi* (Supplementary fig. 2F, G) is characterized by an ornamented process on the outer bone surface.

Palaeobatrachidae

In our sample of Palaeobatrachidae, only one specimen, *P. luedecki*, did not preserve a frontoparietal or parts thereof (Supplementary fig. 3E, see also above). The specimen of *P. grandipes* (Supplementary fig. 3D) retained fragments that can be attributed to a frontoparietal, based on the presence of supraorbital ridges and a partially preserved orbital margin. The remaining specimens possessed well preserved frontoparietals with a clearly visible posterior median process, orbital margins, and supraorbital ridges. The maxilla was generally better preserved than the frontoparietal (Supplementary fig. 3). Two specimens of *Palaeobatrachus sp.* (Supplementary fig. 3B, C) show only the right maxilla, one with a flat pars palatina that extended laterally, and the other with the pars

dentalis. In *P. grandipes* (Supplementary fig. 3D) and *P. luedecki* (Supplementary fig. 3E), the lateral expansion of the pars palatina is visible, along with a dorsoventral expansion in what seems to be the pars facialis.

Trait – SVL relationship

The results of the regression analysis between overall Snout–vent length (SVL) length and microanatomical characteristics (Supplementary fig. 4) showed different outcomes for each of the traits. For compactness (Supplementary Fig. 4A), frontoparietal values showed a weak negative trend with size (slope = –0.074, p = 0.0013), driven primarily by the Non–Pipoidea group, while Pipidae and Palaeobatrachidae exhibited little to no size dependence. Maxilla compactness remained largely unrelated to SVL across all groups (slope = –0.023, p = 0.377). Cross-sectional area (Supplementary fig. 4B) and thickness (Supplementary fig. 4C) generally increased with size in both frontoparietal and maxilla bones, with trends clearest in Non–Pipoidea and Pipidae. Palaeobatrachidae displayed broader variability and wider confidence intervals, reflecting greater uncertainty.

Uncertainty was relatively low for Non-Pipoidea and Pipidae, with consistently narrow confidence bars across the size range. On the other hand, Palaeobatrachidae regression fits were associated with broader intervals and higher uncertainty. Overall, Non–Pipoidea, Pipidae and Palaeobatrachidae generally exhibited positive size-related trends in bone cross-sectional area and thickness, whereas in the compactness, Palaeobatrachidae and Pipidae showed little to no relationship (Additional table 1).

While these patterns are consistent with expected size effects on skeletal dimensions, the limited sample size and grouping of multiple species imply that results should be interpreted cautiously, and pseudo-replication cannot be entirely ruled out as these analyses suggest general patterns rather than definitive species-level conclusions.
