# Supplementary figures and images for: Microanatomy of Dermal Roofing Bones in the Skull of Pipoid Frogs
Source: J Morphol. 2025 Dec 20;286(12):e70107. doi: 10.1002/jmor.70107 (PMC12717838; doi:10.1002/jmor.70107)

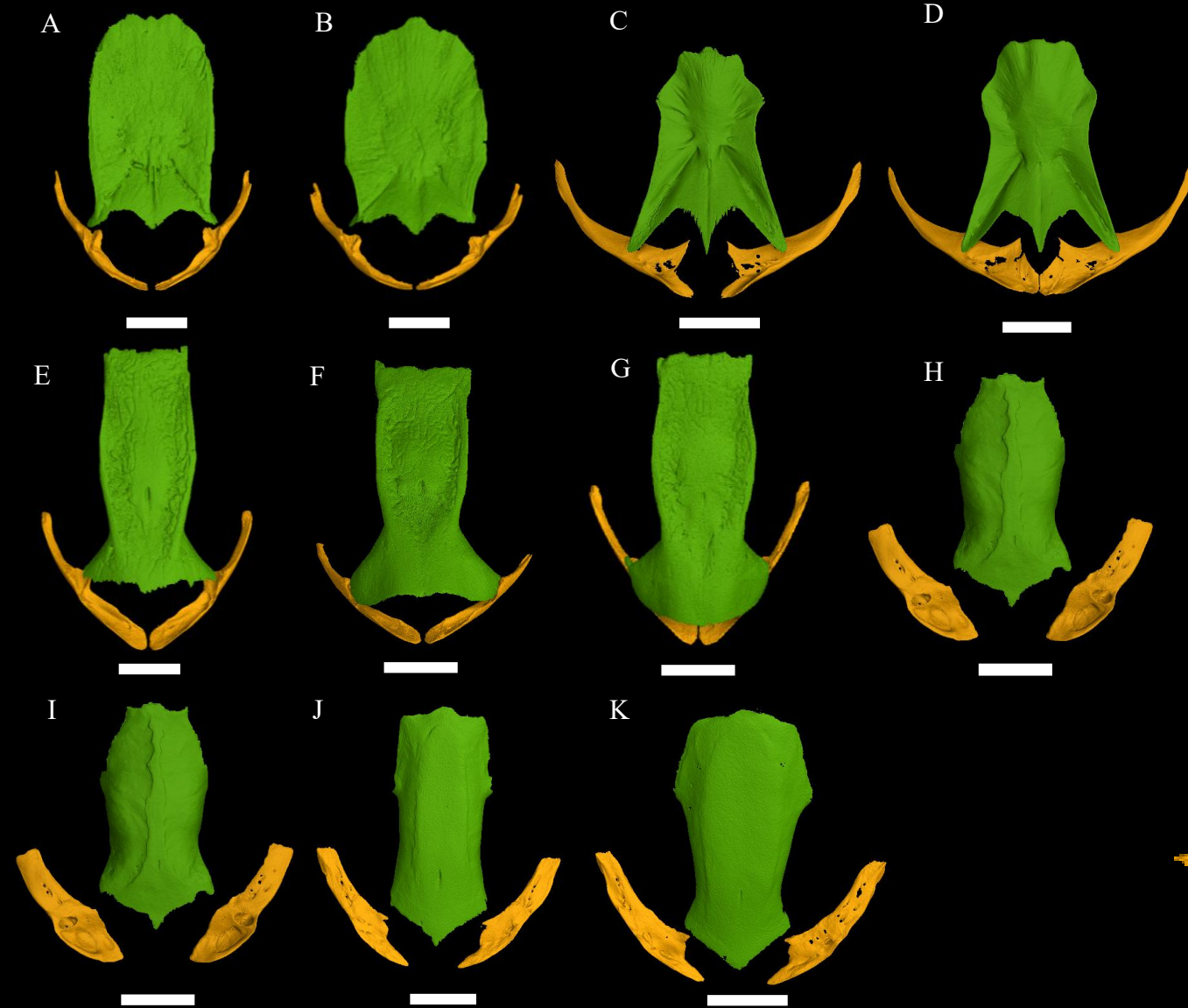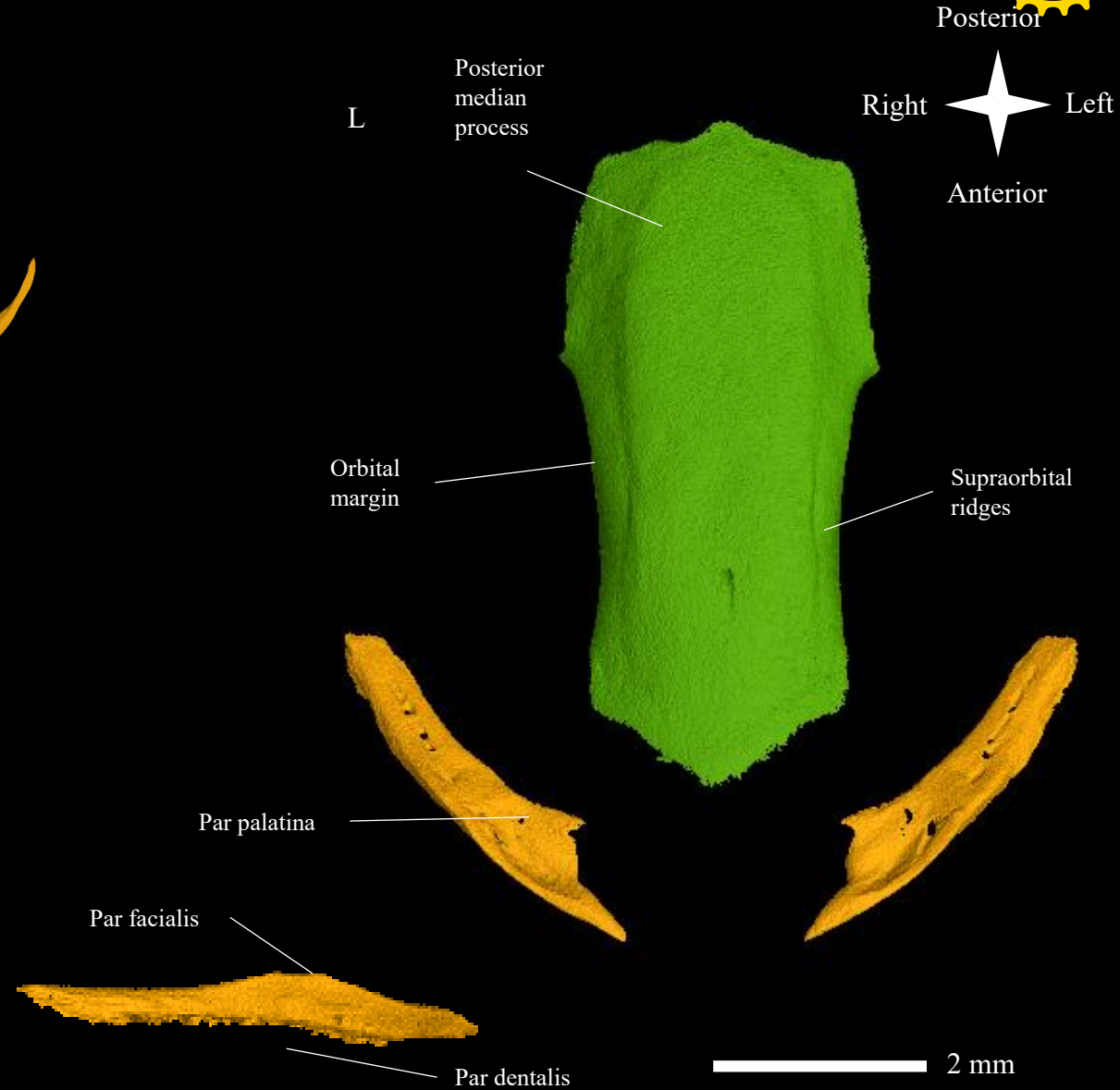

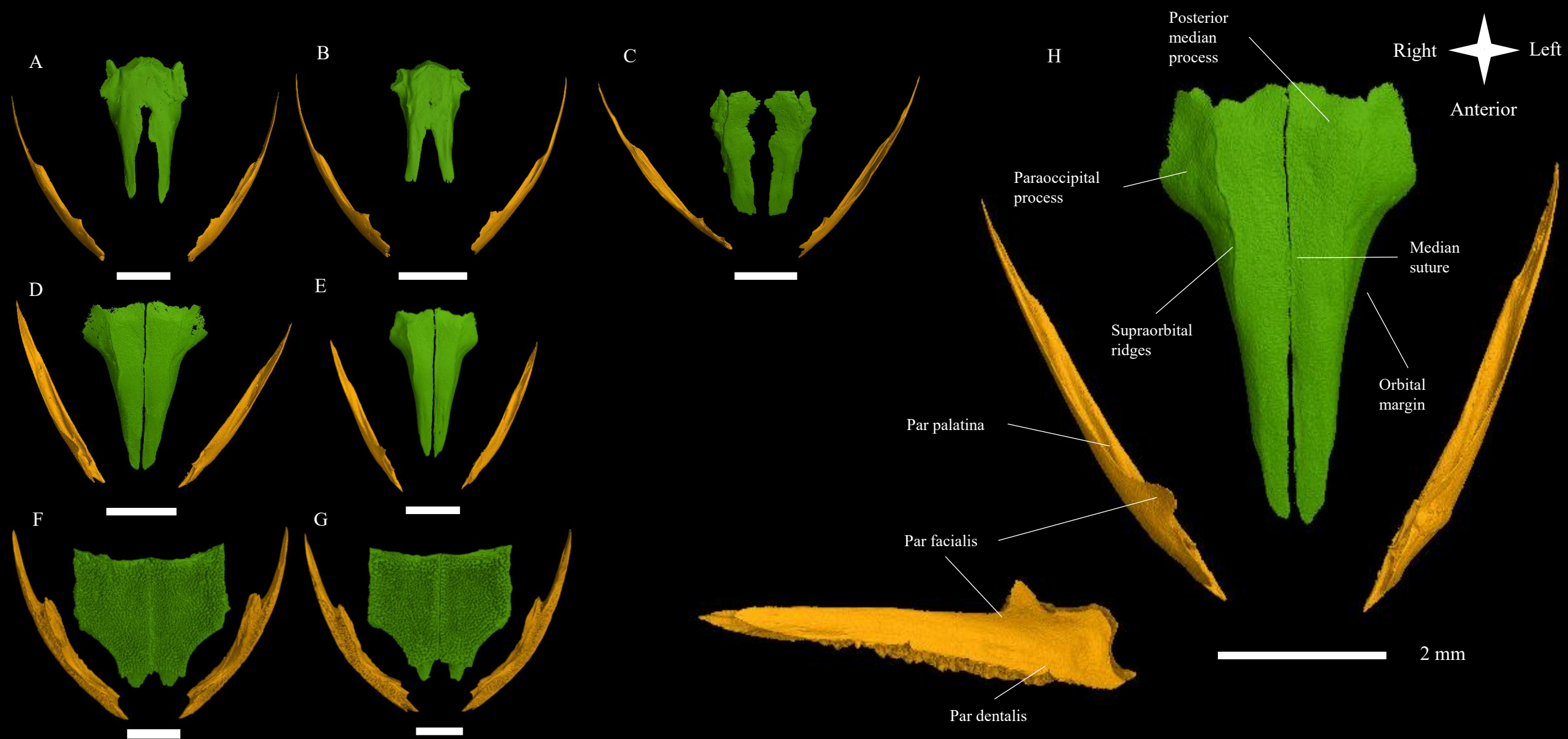

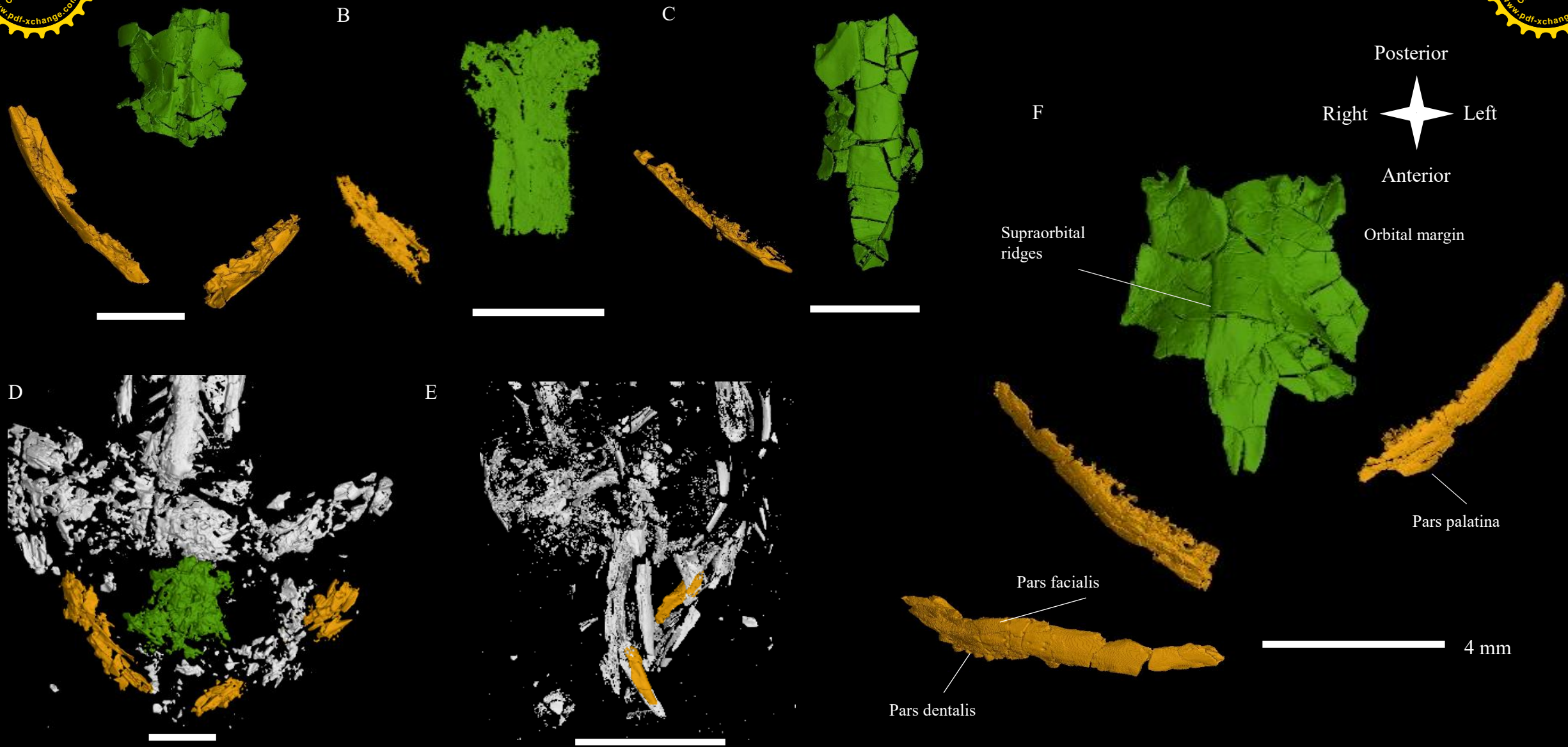

Supplementary figure 4

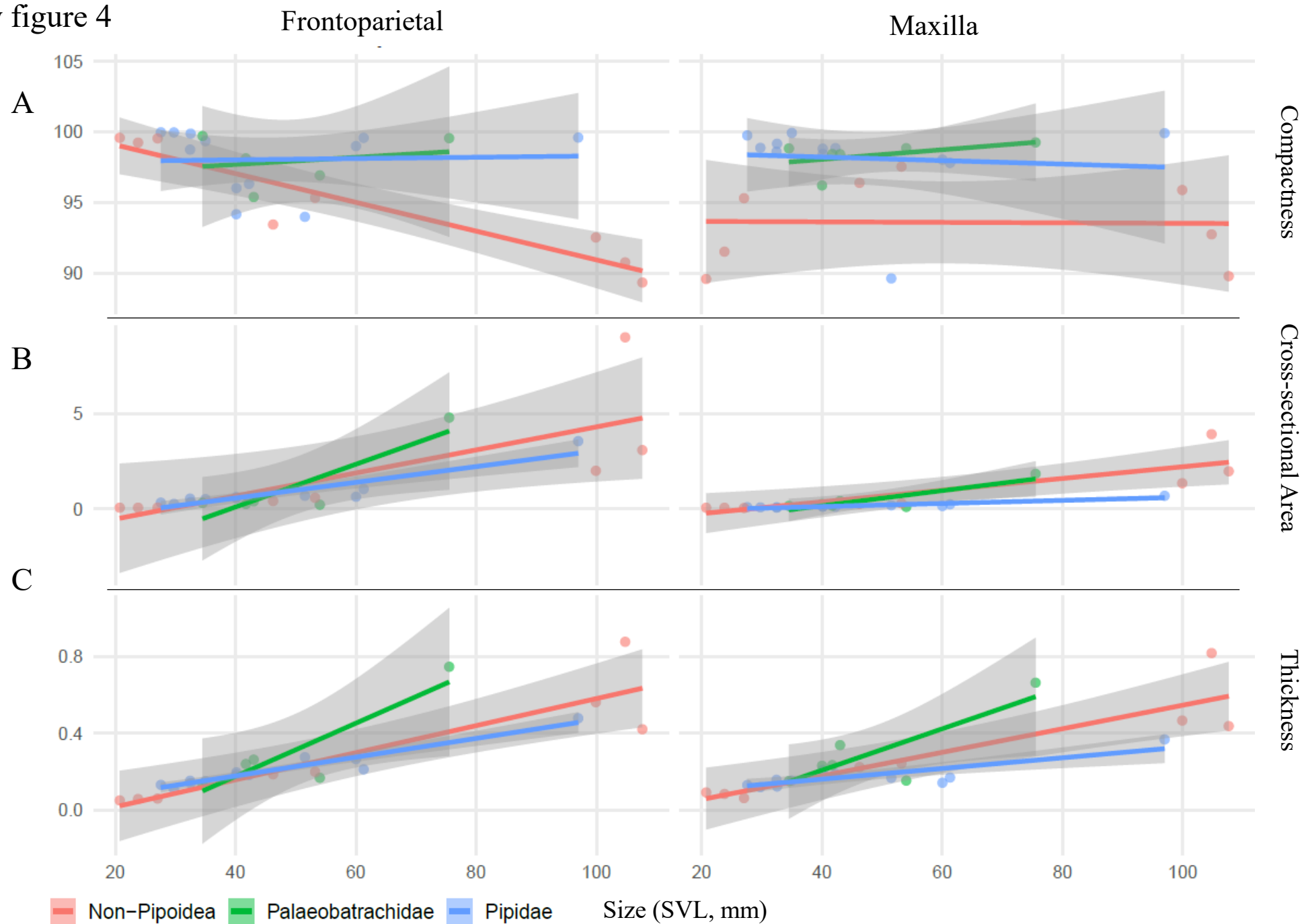

Supplement: Supplementary file 3 — Figures. [file JMOR-286-e70107-s002.pdf]
