# Supplementary material for: Microanatomy of Dermal Roofing Bones in the Skull of Pipoid Frogs
Source: J Morphol. 2025 Dec 20;286(12):e70107. doi: 10.1002/jmor.70107 (PMC12717838; doi:10.1002/jmor.70107)
Supplement: Supplementary file 4 — Figures_legends. [file JMOR-286-e70107-s001.pdf]

Click to BUY NOW! PDF-XChange Editor www.pdf-xchange.com

Click to BUY NOW! PDF-XChange Editor www.pdf-xchange.com

Supplementary figure legends:

Supplementary figure 1. Frontoparietal and maxilla of the Pipidae analysis presented in a dorsal view in living position. A. *Hymenochirus boettgeri* 86837, scale bar 2mm. B. *Hymenochirus boettgeri* 19966, scale bar 2mm. C. *Pipa pipa* 25980a, scale bar 8mm. D. *Pipa pipa* 25980b, scale bar 4mm. E. *Pseudohymenochirus merlini* 90650, scale bar 2mm. F. *Pseudohymenochirus merlini* 90651, scale bar 2,5mm. G. *Pseudohymenochirus merlini* 90652, scale bar 2,5mm. H. *Silurana tropicalis* ZMB86654, scale bar 2,5mm. I. *Silurana tropicalis* 86672, sclae bar 2,5mm. J. *Xenopus laevis* ZMB29204 a, scale bar 2mm. K. *Xenopus laevis* ZMB29204 a, scale bar 2mm. L. *Xenopus laevis* ZMB29204 b, scale bar 2mm with descriptive terminology of frontoparietal and . maxilla in frontal and lateral view.

Supplementary figure 2. Frontoparietal and maxilla of the non-Pipoidea analysis presented in a dorsal view in living position. A. Telmatobius marmoratus ZMB26211, scale bar 3,5mm. B. *Telmatobius marmoratus* ZMB18896, scale bar 5mm. C. *Telmatobius macrostomus* ZMB7700, scale bar 8,5mm. D. *Occidocyga lima* ZMB 91010, scale bar 2mm. E. *Occidocyga lima* ZMB 91009, scale bar 2mm. F. *Calyptocalicephalella gayi* ZMB 26117, scale bar 8mm. G. *Calyptocalicephalella gayi* ZMB 4324, scale bar 8mm. H. Occidocyga lima ZMB 91011, scale bar 2mm with descriptive terminology of frontoparietal and maxilla in frontal and lateral view.

Supplementary figure 3. Frontoparietal and maxilla of the Palaeobatrachidae analysis presented in a dorsal view. A. *Palaeobatrachus sp.* GMH Ce IV-6699-1933, scale bar 4mm. B. *Palaeobatrachus sp.* ZMBAM606, scale bar 5mm. C. *Palaeobatrachus sp.* GMH Ce III-4962-1932, scale bar 4.5mm. D. *Palaeobatrachus grandipes* GMH CeIII1312 1932, scale bar 10mm. E. *Palaeobatrachus luedecki* ZMBMBAM878, scale bar 10mm. F. *Palaeobatrachus sp.* GMH Ce IV-6691-1933, scale bar 4mm.

Supplementary figure 4. Bone traits and size (mm) relationship in the frontoparietal (left) and maxilla (right). Rows correspond to compactness (top), cross-sectional area (middle), and thickness (bottom). Points represent individual specimens. Colored regression lines with shaded areas indicate fitted slopes and 95% confidence intervals for each group: red = Non-Pipoidea, green = Palaeobatrachidae, blue = Pipidae.
